# E46K α-Synuclein Mutation Fails to Promote Neurite Outgrowth by Not Inducing Cdc42EP2 Expression, Unlike Wild-Type or A53T α-Synuclein in SK-N-SH Cells

**DOI:** 10.3390/brainsci15010009

**Published:** 2024-12-25

**Authors:** Hyunja Jung, Seonghan Kim

**Affiliations:** Department of Anatomy, College of Medicine, Inje University, Busan 47392, Republic of Korea

**Keywords:** α-synuclein, neurite outgrowth, Cdc42EP2, Parkinson’s disease, E46K mutation

## Abstract

Background/Objectives: α-Synuclein (α-syn) protein is a major pathological agent of familial Parkinson’s disease (PD), and its levels and aggregations determine neurotoxicity in PD pathogenesis. Although the pathophysiological functions of α-syn have been extensively studied, its biological functions remain elusive, and there are reports of wild-type (WT) α-syn and two missense mutations of α-syn (A30P and A53T) inducing protective neuritogenesis through neurite outgrowth. However, the function of another α-syn mutation, E46K, has not been fully elucidated. Thus, we compared the effect of E46K α-syn with other types to identify the mechanisms underlying neurite outgrowth. Methods: We transfected SK-N-SH cells with WT and mutant (A53T and E46K) α-syn to investigate the effects of their overexpression on neurite outgrowth. Then, we compared the differential effects of α-syn on neurite outgrowth using microscopic analysis, including confocal microscopy. We also analyzed the differential regulation of cell division control 42 effector protein 2 (Cdc42EP2) using real-time quantitative polymerase chain reaction and western blot analysis. Finally, to confirm the implication of neurite outgrowth, we knocked down Cdc42EP2 using small interfering RNA. Results: Unlike WT and A53T α-syn, E46K α-syn failed to promote neurite outgrowth by not inducing Cdc42EP2 and subsequent βIII-tubulin expression. Cdc42EP2 knockdown impaired neurite outgrowth in WT and A53T α-syn transfectants. Conclusions: Our findings suggest that WT and mutant α-syn are linked to Cdc42EP2 production in neuritogenesis, implying α-syn involvement in the physiological function of axon growth and synapse formation. Thus, α-syn may be a potential therapeutic target for PD.

## 1. Introduction

Parkinson’s disease (PD) is the second most common neurodegenerative disease after Alzheimer’s disease. Clinical characteristics include progressive motor dysfunction, such as muscle rigidity, bradykinesia, resting tremor, and other nonmotor symptoms. Lewy bodies (LBs) are the pathological hallmark of PD. These cytoplasmic protein inclusions are present in degenerated dopaminergic neurons in the substantia nigra pars compacta. LBs mainly comprise aggregates of α-synuclein (α-syn), a small protein (140 amino acid residues) that is abundant in neurons, particularly in the synaptic vesicles in the presynaptic nerve terminal and non-neuronal cells, such as glial cells [1,2]. Several α-syn missense mutations (A30P, A53T, E46K, H50Q, and G51D) and multiplication mutations have been identified as genetic causes of familial PD, which makes up 10% of PD cases. Additionally, comprehensive genetic studies have revealed that α-syn is a risk factor for sporadic PD. Although the mechanisms of α-syn toxicity remain poorly understood, it is accepted that aberrant aggregations of α-syn are crucial players in neurodegeneration and that α-syn levels are controlled via the autophagy-lysosome pathway [3,4].

A firm grasp of the normal physiological functions of α-syn is crucial to understanding its role in PD pathogenesis. Although these functions have not been fully elucidated, α-syn has been reported as essential for synaptic vesicle recycling [5], neurotransmitter release, and SNARE complex assembly [6], implying that it plays a neuroprotective role in neurogenesis [7,8,9]. There are reports of both wild-type (WT) and mutant (A30P and A53T) α-syn promoting neurite outgrowth, leading to axon elongation and dendritic arborization, the principal morphological characteristics of neuronal differentiation. Furthermore, α-syn-induced neurite outgrowth was observed in the olfactory bulb and primary neurons during early-phase neuritogenesis [10,11,12,13,14]. Neurite growth is a complex process influenced by extracellular and intracellular signals. Several extracellular signals play crucial roles in regulating neurite growth and guidance. Microtubules are essential for the initial formation and extension of neurites. As neurites develop, microtubules provide essential structural support and guidance [15]. The organization of microtubules helps establish and maintain neuronal polarity, a crucial factor for proper axon and dendrite development. Thus, dynamic neurite outgrowth during development results in a complex neuronal architecture that establishes the functional nervous system and brain. α-Syn facilitates microtubule organization by regulating trophic factor synthesis (brain-derived neurotrophic factor, nerve growth factor, and transforming growth factor-β1) [16,17]. Thus, α-syn-promoted neurite outgrowth is essential for axonal trafficking, a process crucial for synaptic function transportation of synaptic vesicle components by motor proteins, such as kinesin and dynein [18]. Additionally, neurite outgrowth contributes to synapse formation and maintains synaptic plasticity [19,20,21]. However, many studies have contradicted the neuroprotective role of α-syn, reporting it as neurodegenerative, and some studies suggest that α-syn is involved in PD pathogenesis [22,23,24,25,26]. Therefore, the biological functions of α-syn require detailed investigation to understand its pathological mechanisms precisely.

The association between different types of α-syn mutations and specific pathological mechanisms is unclear. Recent publications have suggested that the E46K mutation of α-syn accelerates aggregation and fibril formation. The increased propensity for aggregation is thought to contribute to the early onset and rapid progression of PD with this mutation [27,28]. The E46K mutant α-syn exhibits increased resistance to degradation compared with the WT mutation of α-syn. From another point of view, these findings suggest that α-syn plays a range of roles in neurodevelopment and differentiation [29]. Furthermore, differential expression levels and clearance rates of α-syn contribute to both neurodegeneration and neuroprotection. Based on previous findings, we hypothesized that E46K α-syn could regulate neurite outgrowth differently than other types of α-syn (WT and A53T) based on its toxicity and clearance rate. Here, we attempted to investigate the levels of neurite outgrowth in α-syn-transfected SK-N-SH cells and explore the regulators of α-syn on neuritogenesis to seek novel targeted therapeutics for PD, offering hope for the future.

## 2. Materials and Methods

### 2.1. Cell Culture

Human neuroblastoma cell line (SK-N-SH) (American Type Culture Collection (ATCC), Manassas, VA, USA) was maintained in Dulbecco’s modified Eagle’s medium high glucose (Invitrogen, Carlsbad, CA, USA) supplemented with 2% fetal bovine serum (FBS) (Hyclone, Wilmington, DE, USA), 100 U/mL of penicillin (Sigma-Aldrich, St. Louis, MO, USA), and 100 μg/mL of streptomycin (Invitrogen). Cells were maintained at 37 °C in a humidified incubator with an atmosphere of 5% CO_2_. The culture medium was replaced every two days, and cells were used once they reached more than 80% confluence.

### 2.2. SK-N-SH Cell Differentiation

SK-N-SH cells were seeded in poly-D-Lysine hydrobromide (5 mg/mL) (Sigma-Aldrich)-coated 6-well microplates and were differentiated using a previously reported protocol [30]. The day after the cells reached 70% confluence, cell differentiation was induced with 10 μM *all-trans* retinoic acid (RA) (Sigma-Aldrich) with 1% FBS, and cell culture was maintained for 5 days, with medium changes every other day. On day 5, 50 ng/mL brain-derived neurotrophic factor (BDNF) (Sigma-Aldrich) was supplemented into the cell medium and maintained for an additional five days, refreshing the medium every other day. Cells were maintained below passage 14. Differentiated cellular morphology was monitored with an inverted Eclipse TS100 culture microscope (Nikon, Tokyo, Japan).

### 2.3. Gene Transfection

pcDNA 3.1+ plasmid (Invitrogen) expressing human WT and A53T α-syn were our laboratory stock and prepared as described previously [31]. The E46K mutation was induced by PCR-based mutagenesis and subcloned in the *HindIII-XhoI* sites of a pcDNA3.1+ vector by Cosmogentech, Inc. (Seoul, Republic of Korea). All constructs were confirmed by sequencing. SK-N-SH cells were transfected with pcDNA3.1+-α-syn-WT, pcDNA3.1+-α-syn-E46K, or an empty vector using X-tremeGENE9 transfection reagent (Roche, Vienna, Austria), according to manufacturer’s manual. All these cDNA-harbored plasmids were prepared in endotoxin-free conditions with EndoFree plasmid kits (Qiagen, Valencia, CA, USA), avoiding endotoxin contamination.

### 2.4. Neurite Outgrowth Measurement

To measure the average neurite extension length per cell, we select the neuron-like differentiated cells in phase contrast microscopic images taken with an Olympus CKX41 microscope (Olympus Inc., Tokyo, Japan). The length of neurites was measured using NIH ImageJ v.1.54f with the Fiji plugin (http://imagej.net, (accessed on 3 July 2024)) [32]. The longest neurite of a neuron was measured from the soma to the tip with a segmented drawing tool. Subsequently, the same image was used with the Neurite-J v.1.1 plugin [33]. Statistical analysis was performed with GraphPad PRISM 9.2.0. software (GraphPad Software Inc., San Diego, CA, USA).

### 2.5. Cell Viability and Proliferation Evaluation

The alamarBlue assay (Thermo Fisher Scientific, Walsham, MA, USA) was performed to determine cell viability. The alamarBlue reagent is a resazurin-based solution used as a cell health indicator. The transfected SK-N-SH cells were plated in 96-well plates at a density of 3 × 10^3^ cells/well in triplicate in 200 μL of culture medium and incubated for 72 h. This was followed by treatment with alamarBlue reagents in 20 μL, and samples were incubated at 37 °C. A fluorescence microplate reader (Molecular Devices, San Jose, CA, USA) measured the absorbance at 530 nm/590 nm (excitation/emission) for 72 h. All samples were assessed in triplicate.

### 2.6. Quantitative Real-Time PCR (qRT-PCR)

Total RNA was isolated from SK-N-SH cells using the RNeasy mini kit (Qiagen) and reverse-transcribed to cDNA using the AccuPower RT Premix kit (Bioneer, Daejeon, Republic of Korea). qRT-PCR was performed to analyze gene expression using the SYBR Green TOPreal^TM^ qPCR2X premix (Enzynomics, Daejeon, Republic of Korea) and an ECO real-time PCR machine (Illumina, San Diego, CA, USA). The PCR condition was as follows: 95 °C for 5 min; 40 amplification cycles of 95 °C for 15 s, and 60 °C for 60 s and cooling. Each reaction was run in triplicate with primers for α-syn (forward: 5′-AGAAGCAGCAGGAAAGACAA-3′; reverse: 5′-CTTCCTCAGAAGGCATTTCA-3′), cell division control 42 effector protein 2 (Cdc42EP2) (forward: 5′-GGAGGGGCCTGAAGAAGATG-3′; reverse: 5′-GGG AGC CAG TCTCTGGAATC-3′), βIII-tubulin (forward: 5′-TCAGCGTCTACTACAACGAGGC-3′; reverse: 5′-GCCTGAAGAGATGTCCAAAGGC-3′), and β-actin (forward: 5′-CATCGT GATGGACTCCGGTGAC-3′; reverse: TCAGGTAGTCAGTCAGGTCC-3′).

### 2.7. Western Blot

Total proteins were extracted from the cell lysates using a lysis buffer containing 50 mM Tris-HCl, 150 mM NaCl, 1% NP-40, and 1 mM EDTA and supplemented with a protease inhibitor cocktail (Sigma-Aldrich). The BCA assay determined protein concentrations. In addition, 10% sodium dodecyl sulfate-polyacrylamide gel electrophoresis (SDS-PAGE) was used to separate equal amounts of protein samples, and proteins were subsequently transferred to the nitrocellulose membrane (Millipore, Billerica, MA, USA). The membrane was blocked with 2% milk dissolved in phosphate-buffered saline containing 0.1% Tween-20 (PBST) for 1 h at room temperature. The membranes were probed for 16 h at 4 °C with the following primary antibodies: anti-α-syn antibody (BD Biosciences, Franklin Lakes, NJ, USA, 610787, 1:1000 dilution), anti-Cdc42EP2 antibody (Abnova, Taipei, Taiwan, H00010435-M01, 1:1000 dilution), anti-βIII-tubulin antibody (Santa Cruz Biotechnology, Santa Cruz, CA, USA, sc-8005, 1:1500 dilution) and anti-GAPDH antibody (Sigma-Aldrich, G8795, 1:2500). The blots were washed in PBST buffer and incubated with horseradish peroxidase-conjugated secondary antibodies (Santa Cruz Biotechnology, sc-2005, sc-2357, 1:2500 dilution) for 1 h at room temperature and visualized using enhanced chemiluminescence reagent (ECL) (Advansta Inc., San Jose, CA, USA). The western blot images were gained using the Amersham Image 600 system (GE Healthcare Life Science, Chicago, IL, USA). The protein levels were quantified using the analysis software built into the instrument.

### 2.8. Confocal Microscopy Analysis

SK-N-SH cells, which were transfected with each α-syn (WT, A53T, and E46K) including empty vector control (vehicle), were grown on coverslips coated with 0.01% poly-L-lysine (Sigma) and then fixed for 10 min at room temperature with 4% paraformaldehyde. For permeabilization, 0.1% Triton X-100 in phosphate-buffered saline (PBS) was applied for 1 min and then blocked with the blocking buffer (10% FBS in PBS) for 1 h. Immunofluorescence was processed with an anti-α-syn antibody (1:1000 dilution) and anti-Cdc42EP2 mouse antibody (1:500 dilution), followed by incubation with Alexa Fluor 594 donkey anti-mouse IgG (H+L) conjugated (sc-362278) and Alexa Fluor 488 donkey anti-rabbit IgG (H+L) (sc-516592) (all from Santa Cruz Biotechnology, 1:1500 dilution) for 1 h at room temperature. The LSM510 META confocal laser scanning microscope with an Axioplan 2 imaging system was used to acquire the images (Carl Zeiss, Osfilden, Germany).

### 2.9. Knock-Down with Small Interference RNA (siRNA) Transfection

Knock-down of Cdc42EP2 expression was performed using siRNA oligonucleotides targeting Cdc42EP2 (Bioneer). The siRNA sequences of Cdc42EP2 were 5′-CUGACU UCAUUGAUCUCUA-dTdT-3′ (sense) and 5′-UAGAGAUCAAUGAAGUCAG-dTdT-3′ (antisense). Non-related negative control siRNA (AccuTarget negative control siRNA, Bioneer) was used to confirm the siRNA’s effect. Cells were seeded at 1.5 × 10^5^ cells/well into six-well plates 16 h before transfection, and siRNA oligonucleotides were added at a concentration of 100 nM diluted in 100 μL serum-free DMEM culture medium. Hiperfect transfection reagent (Qiagen) was applied for siRNA transfection and cultured for 6 h. After the culture medium with siRNA complexes was replaced, the second transfection with α-syn expression vectors was performed with X-tremeGENE9. The co-transfected cells were incubated for an additional 72 h to ensure the validity of the siRNA knock-down effects.

### 2.10. Statistical Analysis

All data are presented as mean ± SD of three independent experiments. Statistical significance was determined with Student’s *t*-test or one-way analysis of variance (ANOVA) using GraphPad PRISM 9.2.0. Software (GraphPad Software Inc.). The minimum level of significance was taken if the *p*-value was <0.05.

## 3. Results

### 3.1. E46K α-Syn Mutant Overexpression Attenuated Neurite Outgrowth

**Figure 1:**
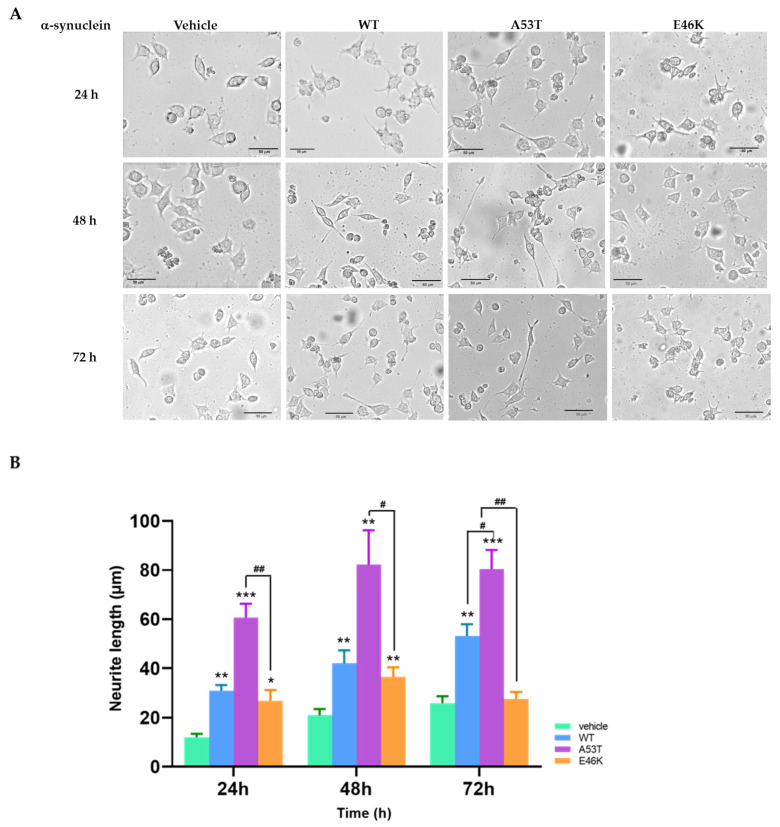
Overexpression of mutant α-synuclein (α-syn) E46K attenuated neurite outgrowth. We transfected SK-N-SH cells with human wild-type (WT) α-syn, A53T or E46K mutant α-syn, or an empty vector using X-tremeGENE 9 DNA Transfection Reagent (Roche) per the manufacturer’s instructions and monitored neurite outgrowth length for 72 h after transfection using an Olympus CKX41 microscope. All images were obtained at 100× magnification. (**A**) After transfection of α-syn transfectants for 24 h, neurite outgrowths of α-syn transfectants were monitored for 72 h. Scale bar: 50 μm. (**B**) Changes in neurite length in each group. The results are presented as means ± standard deviation (SD) for three distinct sets of experiments. The elongated neurite lengths after α-syn transfection were measured using ImageJ-Fiji software (v.1.54f) with the Neurite-J v.1.1 plugin. Differences among each group were compared with the control group using one-way analysis of variance (ANOVA; *** *p* < 0.001). Student *t*-test was used to determine the statistical significance of each group (* *p* < 0.05, ** *p* < 0.01, *** *p* < 0.001, # *p* < 0.05, or ## *p* < 0.01).

We transfected WT α-syn and two missense mutations (A53T and E46K) of α-syn into the human neuroblastoma cell line SK-N-SH to compare their effects on neurite outgrowth promotion. After transfection, we used microscopy to monitor morphologic changes in all transfectants, including the empty vector group (vehicle), for 72 h. At 24 h, the α-syn transfectants showed the initiation of dendritic sprouting from the cell membranes, especially in the WT (2.65 fold), A53T (5.19 fold), and E46K (2.30 fold) α-syn groups compared with the control group (Figure 1A). At 48 h, the sprouting was more prominent (WT: 2.02 fold, A53T: 3.93 fold, and E46K: 1.81 fold). A53T α-syn transfected SK-N-SH cells showed maximum neurite outgrowth (82.17 ± 31.42 μm) at 48 h. At 72 h, A53T α-syn transfectants still maintained the highest neurite outgrowth (3.1 fold, 80.35 ± 17.45 μm), followed by WT α-syn transfectants (2.5 fold) compared with the time control group (vehicle). E46K α-syn transfection had no observable effect on neurite outgrowth compared with the normal growth pattern of the control group (vehicle). We analyzed the changes in neurite length in each group using microscopy and ImageJ-Fiji v.1.54f software (Figure 1B). Notably, compared with the average cell growth and proliferation of the control group, transfection with E46K α-syn did not promote neurite growth during the 72 h incubation period. This stark contrast in the mutants’ effects underscores the importance of our research in elucidating the role of different types of α-syn mutants in neurite growth.

### 3.2. Proliferation of E46K Mutant α-Syn Transfected SK-N-SH Cells

**Figure 2:**
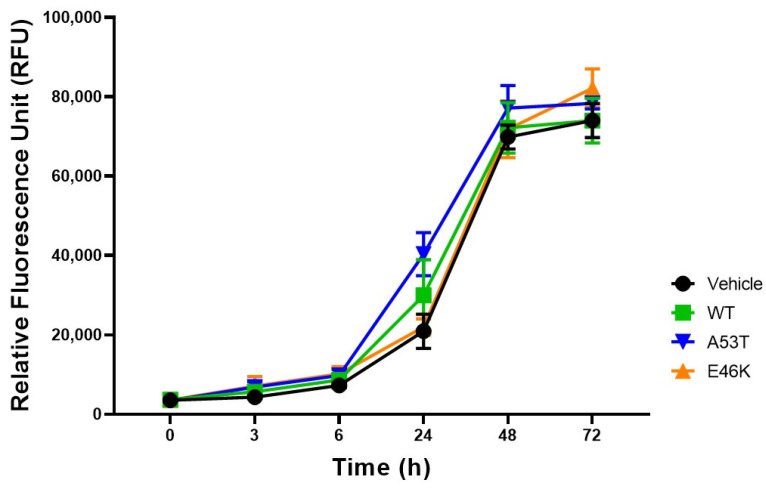
Proliferation of mutant α-syn E46K transfectants. We performed cell proliferation assays using the alamarBlue reagent for 72 h after transfection with pcDNA3.1+-α-syn (WT, A53T, or E46K). The fluorescence of the alamarBlue indicator (excitation, 530 nm; emission, 590 nm) was analyzed using a microplate fluorometer. All samples were prepared in triplicate. Data are presented as means ± SD.

Our findings confirmed that E46K α-syn significantly attenuated neurite outgrowth compared with WT or A53T α-syn. To ascertain whether the attenuated neurite elongation observed in the E46K α-syn transfectant was attributed to enhanced cell death or impaired cell proliferation (unlike WT or A53T α-syn), we performed alamarBlue assays to evaluate cell proliferation/viability (Figure 2). We introduced the alamarBlue reagent to the cell culture medium at 24 h after transfection and continued monitoring for another 72 h at each time interval (0, 3, 6, 24, 48, and 72 h). There was no indication of blocked cell proliferation at 72 h after adding alamarBlue to the culture medium, and all transfected SK-N-SH cell groups exhibited similar patterns of growth and proliferation. These findings indicated that the reduced neurite outgrowth of E46K α-syn was not due to the reduced cell viability but primarily due to the effect of E46K α-syn, a finding that underscores the importance of our research.

### 3.3. Cdc42EP2 Negatively Regulated Neurite Outgrowth by Downregulating βIII-Tubulin in E46K α-Syn Transfectants

**Figure 3:**
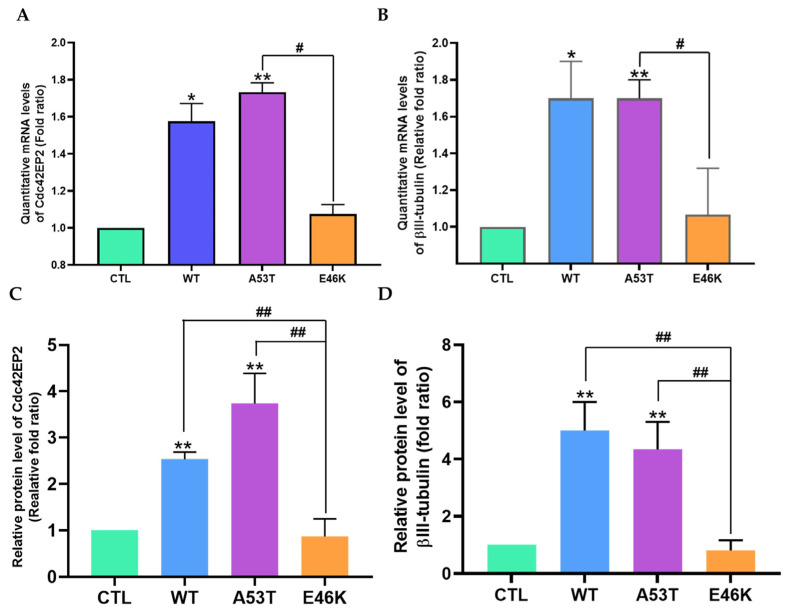
Cell division control 42 effector protein 2 (Cdc42EP2) negatively regulated neurite outgrowth by downregulating βIII-tubulin in E46K α-syn transfectants. (**A**) Quantitative analysis of Cdc42EP2 mRNA expression. SK-N-SH cells were seeded into six-well plates (1 × 10^5^ cells/well) and cultured for 16 h to achieve attachment and confluency. After transfection with each pcDNA3.1+-α-syn (WT, A53T, and E46K), the cells were maintained at 37 °C in an atmosphere of 5% CO_2_ for 24 h. Then, total RNA was isolated for cDNA synthesis, followed by real-time quantitative PCR using SYBR Green TOPreal^TM^ qPCR PreMIX (Enzynomics) on an Eco Real-Time PCR System (Illumina). Data are presented as means ± SD from four independent experiments. (**B**) Quantitative analysis of βIII-tubulin gene expression using the same techniques and statistical analyses as described in Figure 3A. (**C**) Western blot analysis of Cdc42EP2 protein levels in α-syn transfectants, including the empty vector group (control). (**D**) Western blot analysis of βIII-tubulin levels in α-syn transfectants, including the empty vector group (control). After sodium dodecyl sulfate-polyacrylamide gel electrophoresis of prepared cell lysate samples and transferring the proteins onto membranes, western blotting was performed using specific antibodies for human α-syn, Cdc42EP2, or βIII-tubulin. Figure S1 presents representative blots from three independent experiments. Data are presented as means ± SD for three independent experiments performed in triplicate. Analysis was performed using one-way ANOVA (* *p* < 0.05, ** *p* < 0.01) and Student *t*-test (# *p* < 0.05, ## *p* < 0.01).

We next investigated the regulator of E46K α-syn-induced neurite outgrowth. Gene profile screening results showed that it was linked to microtubule assembly and neuritogenesis. We chose the cell division control 42 effector protein 2 (Cdc42EP2) gene from the pool of potential candidate genes due to observed variations in expression levels in the E46K α-syn transfectants compared with the WT and A53T α-syn transfectants. At 24 h post-transfection, we used quantitative real-time PCR to evaluate the Cdc42EP2 mRNA levels in the transfected cells, revealing significant increases in the WT and A53T α-syn transfectants (68% ± 3.2% and 80% ± 2.1%, respectively). In contrast, the E46K α-syn transfectants exhibited a mere 7% ± 1.5% increase compared with SK-N-SH cells transfected with empty vectors (control) (Figure 3A). The levels of Cdc42EP2 expression correlated with neurite outgrowth, and this novel finding prompted us to investigate the role of α-syn in microtubule formation through Cdc42EP2 regulation. After screening other associated candidates, we decided to investigate the target gene of Cdc42EP2. We observed that the expression of an alternative microtubule component, βIII-tubulin, was increased in the WT (79% ± 4.1%) and A53T (70% ± 2.5%) α-syn transfectants but not in the E46K α-syn transfectants (Figure 3B). These findings were crucial to understanding the complex interplay of genes and proteins in regulating neurite outgrowth.

We then investigated the effect of α-syn transfection on the protein levels of Cdc42EP2 and βIII-tubulin by quantitatively analyzing the differential protein expression of Cdc42EP2 in each transfectant group (Figure 3C) using western blotting. Briefly, cell lysates were prepared after 48 h of transfection to ensure adequate time for protein expression. Densiometric analysis of the western blotting results revealed upregulated Cdc42EP2 protein expression in the WT (153% ± 1.5%) and A53T (273% ± 3.8%) α-syn transfectants, indicating the potential involvement of α-syn in regulating Cdc42EP2 expression. In contrast, there was no significant increase in Cdc42EP2 protein expression in the E46K α-syn transfectants compared with the control group (Figure 3C). The discovery of the induction of the microtubule protein unit, as evidenced by βIII-tubulin protein expression in WT (400% ± 3.0%) and A53T (330% ± 2.8%) α-syn transfectants, highlighted the unique nature of our research. In contrast, E46K α-syn overexpression decreased the levels of βIII-tubulin protein (Figure 3D). Thus, our findings demonstrated that unlike WT and A53T α-syn, E46K α-syn could not induce Cdc42EP2 and subsequent βIII-tubulin expression. Among the proteins implicated in microtubule assembly, tau protein, responsible for microtubule stabilization in axons, did not exhibit any notable alterations following α-syn transfection (Figure S1).

Our findings strongly suggested that Cdc42EP2 induction was crucial for α-syn-induced neurite outgrowth. Furthermore, they indicated a direct link between the observed increase in Cdc42EP2 levels and βIII-tubulin expression, which is crucial for microtubule formation in dendrites and axons.

### 3.4. α-Syn-Induced Cdc42EP2 Expression Regulated Neurite Outgrowth in SK-N-SH Cells

**Figure 4:**
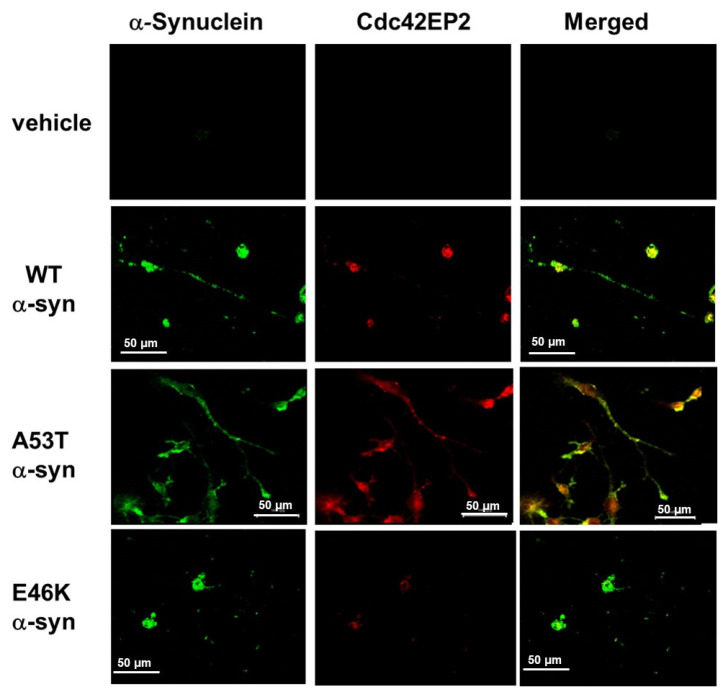
α-Syn-induced Cdc42EP2 expression regulated neurite outgrowth in SK-N-SH cells. Confocal microscopy analysis of SK-N-SH cells transfected with WT, A53T, and E46K α-syn, including empty vector control (vehicle), revealed the correlation of Cdc42EP2 with the mutations of α-syn. Immunofluorescent staining was performed using Alexa Fluor 488-conjugated secondary antibody (green). α-Syn-induced Cdc42EP2 was visualized using Alexa Fluor 594 staining (red). Immunofluorescent images were captured using an LSM510 META confocal microscope with an Axioplan 2 imaging system (Carl Zeiss). Scale bar: 50 μm.

We used confocal laser scanning microscopy to examine the intracellular expression and localization of Cdc42EP2 induced by each type of α-syn (WT, A53T, and E46K). WT and A53T α-syn exhibited a remarkable increase in neurite outgrowth compared with E46K α-syn (Figure 1). Furthermore, our investigations unveiled a significant correlation between α-syn and Cdc42EP2 expression and their influence on neurite outgrowth in SK-N-SH cells. We also found that overexpressed α-syn induced Cdc42EP2 expression. In A53T α-syn transfectants, we observed elongated neurites with α-syn and Cdc42EP2 expression, as seen in the cytoplasm. WT α-syn also showed Cdc42EP2 signals in neurites but were relatively lower than A53T α-syn. Our study findings demonstrated that the coexpression of α-syn and Cdc42EP2 was critical for stimulating neurite outgrowth. The inability of E46K α-syn to induce Cdc42EP2 prevented neurite growth (Figure 4). Thus, our study emphasizes that α-syn-induced Cdc42EP2 expression is crucial for regulating neurite outgrowth.

### 3.5. Cdc42EP2 Knockdown Abrogated α-Syn-Induced Neurite Outgrowth

**Figure 5:**
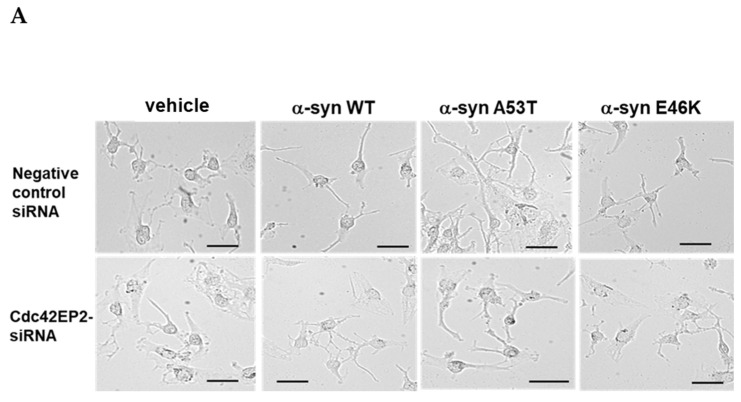

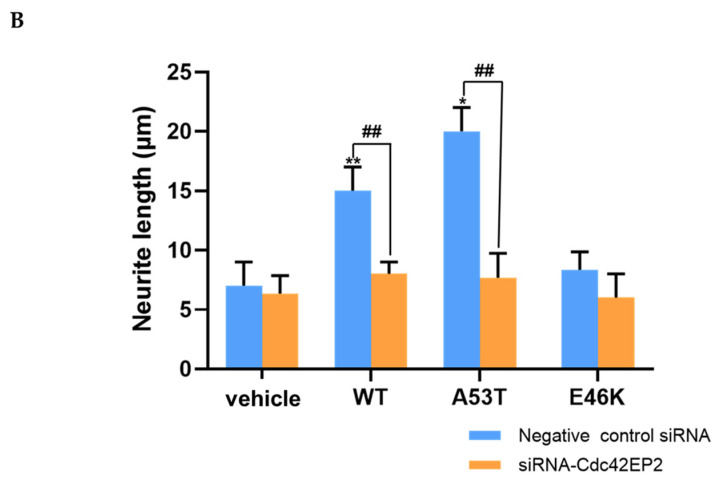
Cdc42EP2 knockdown abrogated α-syn-induced neurite outgrowth. Small interfering RNA (siRNA) targeting Cdc42EP2 was transfected into SK-N-SH cells. Unrelated siRNA was used as a negative control. Following a 6-hour interval, we performed cotransfection with secondary α-syn expression cDNA (WT, A53T, or E46K). An empty vector was used as the control. (**A**) At 72 h, the peak point of siRNA action, we assessed morphological changes in the cotransfected SK-N-SH cells using microscopy. Scale bar: 50 μm. (**B**) We performed three independent experiments to evaluate the changes in neurite lengths of the transfectants. Results are presented as mean ± SD. Statistical analysis was performed using one-way ANOVA (* *p* < 0.05 and ** *p* < 0.01) and Student *t*-test (## *p* < 0.01).

Our confirmation of the correlated expression of α-syn and Cdc42EP2 established Cdc42EP2 as the critical regulator of α-syn-induced neurite outgrowth. To validate this hypothesis, we cotransfected SK-N-SH cells with α-syn (WT, A53T, or E46K) and small interfering RNA (siRNA) targeting Cdc42EP2 to inhibit its expression. Before α-syn transfection, we transfected the cells with siRNA targeting Cdc42EP2 for 6 h. To achieve maximum siRNA effectiveness, we maintained the transfectant for 72 h. The western blot results validated the reduction in Cdc42EP2 expression, indicating that the siRNA had effectively suppressed protein expression (Figure S2). We also transfected SK-N-SH cells with an unrelated negative control siRNA to nullify any potential siRNA influences and facilitate a comparison of the target gene (Cdc42EP2). Microscopic analysis revealed that Cdc42EP2 knockdown abrogated neurite outgrowth in both WT and A53T α-syn transfectants, similar to the results observed in the control group (Figure 5A). Neurite outgrowth remained unaffected when Cdc42EP2 expression was induced using the unrelated negative control siRNA, and only α-syn-associated neurite outgrowth was observed. The total lengths of neurites were shorter in cells cotransfected with α-syn and siRNA than α-syn transfected only. However, the overall trend of neurite outgrowth remained similar. Analysis of the neurite lengths confirmed our hypothesis that α-syn-induced Cdc42EP2 was a crucial regulator of neurite outgrowth (Figure 5B).

## 4. Discussion

Since its discovery, α-syn has attracted attention as a possible genetic pathogenic agent of familial PD. Because it is one of the principal components of LBs, which are pathological hallmarks of PD, research has focused on understanding the pathogenic mechanism of α-syn in neurodegenerative diseases, and PD in particular. Over the last decades, there have been many reports linking the levels of intracellular α-syn protein with neurotoxicity, leading to cell death via apoptosis and the activation of autophagic pathways [3,8,34,35]. PD pathogenesis involves the death of dopaminergic neurons, thereby reducing dopamine release [26]. α-Syn plays a role in cellular differentiation processes, particularly neurons; its precise effects and mechanisms are still being elucidated and may vary depending on the cell types. α-Syn controls neuronal differentiation negatively in neuronal progenitor cells and dopaminergic neurons [36]. However, α-syn promotes the differentiation of erythrocytes and osteoblasts. Studies on T cells have shown that α-syn promotes the differentiation of T helper 17 (Th17) cells while impairing the function of regulatory T cells (Tregs) [37]. Although our understanding of the precise pathogenic mechanisms of α-syn is limited, its neurotoxicity may be related to the destabilization of microtubule assembly in neurites [24,25,38,39]. Many studies have examined the involvement of this protein in neurodegenerative pathology. However, the biological functions of α-syn under normal physiological conditions remain elusive. It is believed that α-syn plays a role in synaptic plasticity and promotes neurotransmitter release. Thus, elucidation of the role of α-syn in the nervous system is crucial to understanding the pathogenic mechanisms of α-syn. Some studies have investigated α-syn-induced neurite outgrowth. These reports, including our study, have led to controversy over the functional role of α-syn in neuronal differentiation and neuritogenesis.

We investigated the differential effect of α-syn on neurite outgrowth. The functional effect of α-syn on neuritic integrity remains under debate, even though most studies have suggested that neurotoxicity impairs neurogenesis. Furthermore, it was reported that α-syn enhanced neurite outgrowth in olfactory and glutaminergic neurons. Nineteen α-syn missense mutations have been identified, among which A30P, A53T, E46K, H50Q, and G51D are well-known [40,41,42]. Most studies have investigated WT α-syn and the two missense mutants A30P and A53T. The E46K α-syn mutation was reported to drive fibril formation and trigger the macroautophagy pathway [43,44,45]. However, its effects on neurite outgrowth remain unknown, leading us to investigate E46K α-syn compared to other α-syn mutants.

In our study, contrary to WT and A53T α-syn, E46K α-syn overexpression did not promote neurite outgrowth. Importantly, this is the first report of E46K α-syn having an opposite effect on neurite outgrowth compared with other types of α-syn. The effect of E46K α-syn on neurite outgrowth was suspected due to reports of the accelerated aggregation of E46K α-syn, which induced neurotoxicity. However, no significant differences in the proliferation of E46K-transfected neuroblastoma cells were observed compared with WT and A53T α-syn, and there was even a slight increase in the proliferation rate (<10%). This led us to conclude that the neurotoxicity of E46K α-syn was unrelated to the suppression of neurite outgrowth. These results are also consistent with reports that no overt loss of dopaminergic neurons or dopamine deficiency was found in the E46K α-syn rat model [46]. The detailed differential mechanism of the E46K α-syn function still requires elucidation. Because α-syn sequence analysis has not yielded any information about functional differences among missense mutations, the intrinsic nature of α-syn remains undetermined [47,48]. Understanding these mechanisms provides insights into how α-syn mutations lead to neuronal dysfunction and death in synucleinopathies.

We searched for extrinsic factors of neurite outgrowth that could potentially be regulated by α-syn. When screening microtubule-associated proteins, we identified Cdc42EP2 as a crucial regulator of neuritogenesis. Cdc42EP2 is a member of the Cdc42 effector proteins (CEP1~CEP5) family, also known as the Borg family because it binds to Rho GTPase, engages with Cdc42, and contains the Cdc42/Rac interactive binding motif. CEPs, including Cdc42EP2, function as downstream effectors of Cdc42, promoting actin filament assembly and subsequent induction of cellular processes [49,50]. This leads to directed protrusion of the membrane at specific sites [51]. Cdc42EP2 has been identified to interact with α-syn using pull-down assays in protein arrays, and it co-localizes with α-syn aggregates in brain sections based on immunostaining, strongly suggesting binding in vivo [52].

Our study revealed that the overexpression of WT and A53T α-syn in SK-N-SH neuroblastoma cells caused neurite outgrowth accompanied by increased levels of Cdc42EP2 expression. In contrast, E46K α-syn overexpression did not induce Cdc42EP2 expression, implying that E46K α-syn inhibits Cdc42EP2 expression and interferes with neurite extension. Confocal microscopic analysis showed that WT and A53T α-syn upregulated Cdc42EP2, which appeared together in cell bodies and extended neurites. The concurrent expression of α-syn and Cdc42EP2 suggest that Cdc42EP2 is a significant regulating target for α-syn-mediated neurite outgrowth. Since Cdc42EP2 was present in extended neurites, we could conclude that axonal trafficking transported it, and it regulated neurite outgrowth along with α-syn. We found that E46K α-syn did not promote neurite outgrowth, possibly by failing to induce the expression of Cdc42EP2, a primary target of α-syn. This effect differs from other α-syn types, such as WT and A53T. The selective reduction of Cdc42EP2 expression by specific siRNAs confirmed our hypothesis of the role of Cdc42EP2 in neurite outgrowth and its reciprocal relationship with E46K α-syn. The alamarBlue assay and siRNA studies showed that the variations in Cdc42EP2 expression by α-syn did not cause differences in cell proliferation, suggesting that the degree of neurite outgrowth did not arise from cell proliferation. The mechanisms of action of Cdc42EP2 in cell differentiation processes affecting neurite outgrowth need to be elucidated in further investigations.

We chose to investigate βIII-tubulin as a specific target of α-syn-regulated Cdc42EP2 expression from the gene profile screening results because it potentially impacted microtubule assembly in neurites. Briefly, βIII-tubulin is a microtubule element found exclusively in neurons, making it a biomarker of neuronal cell differentiation and development [53,54]. We found a correlation between Cdc42EP2 and βIII-tubulin upregulation and the stimulation of neurite outgrowth by WT and A53T α-syn. We concluded that the α-syn-induced neurite outgrowth pathway included Cdc42EP2-regulated βIII-tubulin expression.

The N-terminal amino acid sequence of human α-syn contains six KTKEGV-type repeats, and most missense mutations occur in these regions. However, sequence analysis has proven to be inadequate to determine the functional differences among α-syn mutants, leaving a significant gap in our current understanding that warrants investigation. E46K α-syn was reported to show enhanced toxicity compared with other α-syn types. However, from the point of view of neurite outgrowth, the effect of E46K is not due to its toxicity. Thus, the differential effects of E46K α-syn still remain obscure, and investigating the specific functions of E46K α-syn in neurite growth compared to other α-syn types is crucial. Further studies are necessary to elucidate these interactions among the α-syn mutants, Cdc42EP2, and βIII-tubulin, in sequence with other associated proteins that may modulate those actions.

## 5. Conclusions

Our study unveils novel insights into the relationship between E46K α-syn mutation and neurite outgrowth. We demonstrated that unlike WT α-syn and other α-syn mutations, the E46K mutation of α-syn did not promote neurite outgrowth because it failed to induce Cdc42EP2 and subsequent βIII-tubulin expression. This study proposes that these two proteins are primary targets of α-syn during neurite outgrowth formation. While the controversy on the role of α-syn in neurite outgrowth continues, our data align with the conclusions of other studies on α-syn-induced neurite outgrowth. The links between the physiological functions and pathological mechanisms of α-syn are still elusive. Studying α-syn and its mutations provides insight into their roles in forming a complex nervous system and its pathological involvement. Our findings may contribute to identifying innovative targets for developing promising therapeutics that reduce α-syn levels, among other functions.

## Data Availability

Data are available in a publicly accessible repository. The original data presented in the study are openly available in Figshare at https://doi.org/10.6084/m9.figshare.27865353 (accessed on 20 November 2024).

## Supplementary Materials

The following supporting information can be downloaded at: https://www.mdpi.com/article/10.3390/brainsci15010009/s1, Figure S1: Western blot analysis of SN-N-SH cells with α-syn overexpression; Figure S2: Knock-down of Cdc42EP2 expression with siRNA in α-syn transfectants.
