# Supplementary material for: E46K α-Synuclein Mutation Fails to Promote Neurite Outgrowth by Not Inducing Cdc42EP2 Expression, Unlike Wild-Type or A53T α-Synuclein in SK-N-SH Cells
Source: Brain Sci. 2024 Dec 25;15(1):9. doi: 10.3390/brainsci15010009 (PMC11763803; doi:10.3390/brainsci15010009)
Supplement: Supplementary file 1 [file brainsci-15-00009-s001.zip › brainsci-3357138-supplementary.pdf]

Supplementary Figures

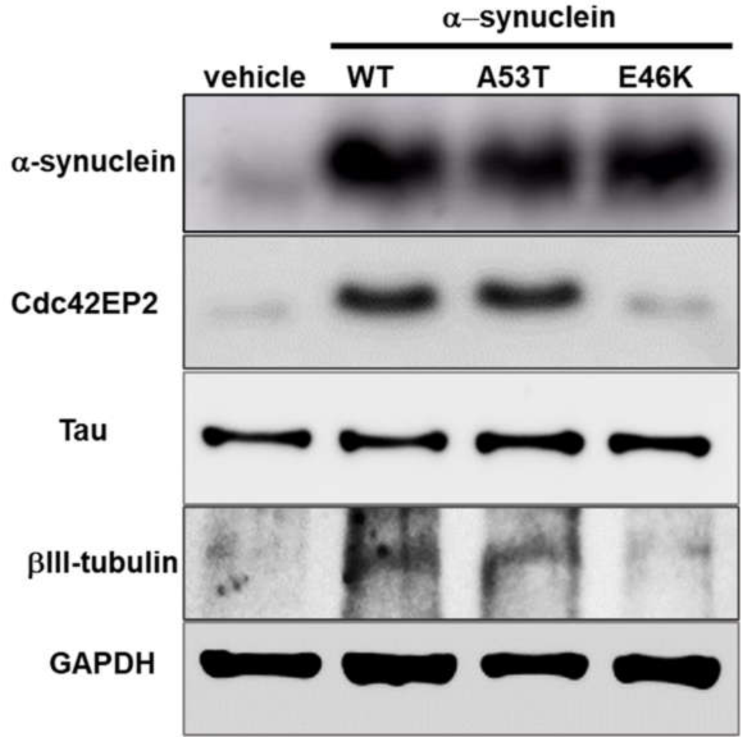

Figure S1: Western blot analysis of α-syn overexpressed SN-N-SH cells

The protein level changes after transfection with α-syn (WT, A53T, and E46K) were analyzed using Western blot analysis, as shown in **Figure 3C** and **3D** through densitometric analysis. The transfectants were cultured for 48 h to ensure optimal protein expression. SK-N-SH human neuroblastoma cells transfected with α-syn exhibited elevated expressions of Cdc42EP2 and βIII-tubulin. Another crucial component of the microtubule, tau protein, showed no significant variation in protein level.

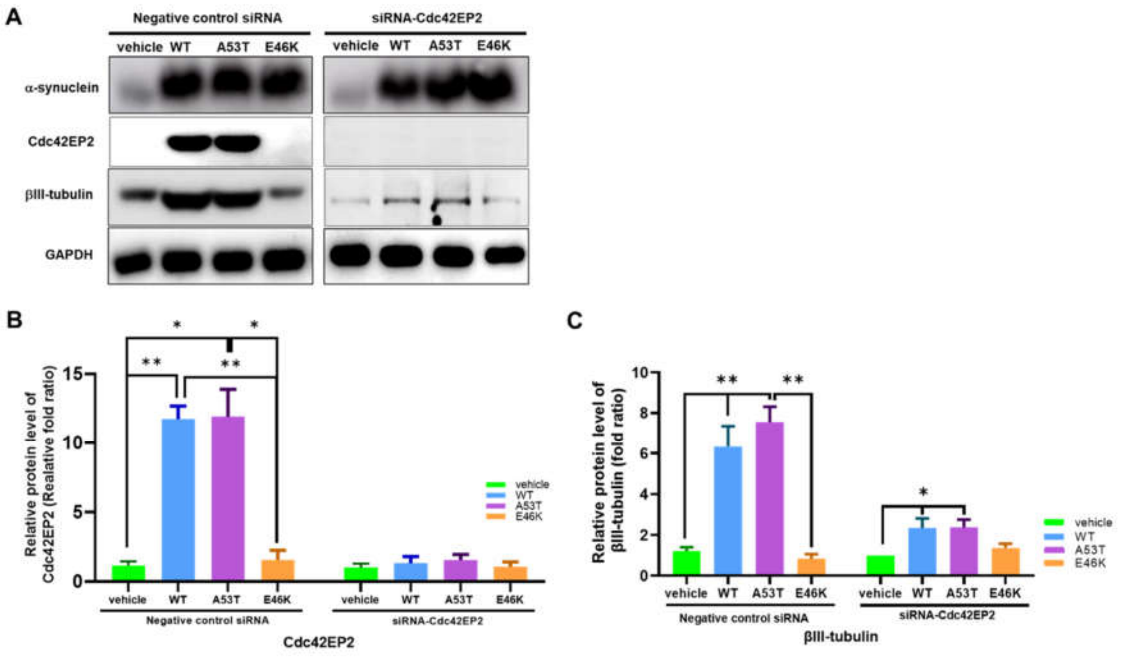

Figure S2: Knock-down of Cdc42EP2 expression with siRNA in α-syn transfectants

After introducing Cdc42EP2-targeted siRNA, co-transfection with each α-syn was performed in SK-N-SH cells. (A) Western blot analysis of transfectants showed the effect of α-syn-induced Cdc42EP2. To mitigate the influence of siRNA, a negative control comprising non-related siRNA was used and subsequently compared. The targeted siRNA-mediated inhibition of Cdc42EP2, Cdc42EP2, and βIII-tubulin decreased protein levels, indicating the downstream impact of Cdc42EP2 on βIII-tubulin. By knocking down Cdc42EP2, we confirmed the relationships and observed the effect on neurite outgrowth, as illustrated in **Figure 5**. (B) Relative protein expressions of Cdc42EP2 of western blot analysis (A) were analyzed in fold ratio. (C) Relative protein expression of βIII-tubulin in each transfectant was analyzed in fold ratio. Data are presented as means±SD for three independent experiments. Statistical analysis was performed using student *t*-test (*$p < 0.05$, **$p < 0.01$).
